# Effectiveness of different molecular forms of *C. histolyticum* class I collagenase to recover islets

**DOI:** 10.1080/19382014.2017.1365996

**Published:** 2017-09-21

**Authors:** Michael L. Green, Andrew G. Breite, Caleb A. Beechler, Francis E. Dwulet, Robert C. McCarthy

**Affiliations:** VitaCyte LLC, Indianapolis, IN, USA

**Keywords:** Clostridium histolyticum, class I, class II, collagenase, collagen degradation activity, islet isolation, porcine islet, tissue dissociation

## Abstract

One factor that may contribute to variability between different lots of purified collagenase to recover islets is the molecular form of *C. histolyticum* class I (C1) collagenase used in the isolation procedure. Two different enzyme mixtures containing C1, class II (C2) collagenase and BP Protease were compared for their effectiveness to recover islets from split adult porcine pancreas. The same enzyme activities per g trimmed tissue were used for all isolations with the only difference being the mass of C1 required to achieve 25,000 collagen degradation activity U/g tissue. The results show no differences in performance of the two enzyme mixtures. The only significant difference is 19 fold more truncated C1 was required to achieve the same result as intact C1.

Collagenase has been recognized as a critical variable for isolation of human islets prior to their subsequent use in therapeutic cell transplantation.^1,2^ Collagenase reliability improved when the enzymes responsible for islet recovery were identified, purified, and incorporated into products designed to increase human islet yields.^3^ Enzyme mixtures containing high amounts of purified *Clostridium histolyticum* collagenase, supplemented with small amounts of neutral protease gave human islet yields superior to those obtained when using crude collagenase.^4,5^ After > 10 years of routine use, several reports indicated the instability of these purified enzymes on storage or inconsistent results between different lots of product.^6-8^ In one report, results indicated sub-optimal performance was associated with changes in biochemical characteristics of collagenase but there was no explanation why islet recovery declined.^6^

Knowledge of the structure-function of *C. histolyticum* collagenase and application of this information provides an explanation for the suboptimal performance of some lots of purified enzymes in human islet isolation. The brief overview below provides essential information on the different classes of *C. histolyticum* collagenase and how different molecular forms of collagenase with neutral protease are responsible for releasing islets from pancreatic tissue. This knowledge is applied in design of an experiment comparing the efficiency of different molecular forms of class I collagenase to recover porcine islets. The focus of this communication is reporting results from these experiments and discussing their implications for isolating porcine or human islets for clinical use.

Collagenases are defined by their unique ability to degrade the triple alpha helical amino acid chain structure of native collagen. This monomeric structure self assembles into larger fibril or fiber structures.^9^ The two classes of *C. histolyticum* collagenase are identified by difference in gelatinase and peptidase activities. Class I (C1) has strong gelatinase activity (i.e., gelatin substrate) but low peptidase activity (i.e., Pz peptide or FALGPA substrate) whereas the converse is true for class II (C2) collagenase.^10^ These enzymes appear to work synergistically with protease to degrade native collagen in the extracellular matrix.^11^ C2 collagenase plays a major role in recovering rodent islets^12,13^ but both classes of collagenase appear to be required for isolating islets from porcine or human pancreata.^14,15^

Subsequent studies showed each class of collagenase was expressed from single copy genes and translated into a full length protein.^16^ C1 or C2 collagenase has four protein domains: an amino terminal catalytic domain, one or two linking domains (function unknown) and one or two collagen binding domains. Functionally active collagenase, defined by the ability to degrade native collagen, must contain a catalytic domain and at least one collagen binding domain. The 3 functionally active forms of this enzyme are intact C1 (C1_116 kDa_) containing a catalytic domain, a linking domain, and two collagen binding domains; intact C2 (C2_114 kDa_) containing a catalytic domain, two linking domains, and one collagen binding domain; and truncated C1 (C1_100 kDa_) containing three domains. This shorter enzyme only has one collagen binding domain. The other carboxy terminal domain is lost likely by proteolysis of 24 amino acid random coil link that connects the two collagen binding domains on intact C1.^17^

A hypothetical mechanism of how collagenase and neutral protease release cells from the extracellular matrix assumes collagenase's collagen binding domain must initially bind to a site on the triple alpha helical protein chain, a characteristic of all forms of native collagen.^16^ Once bound, collagenase's catalytic domain can cut the collagen, followed by unbound collagenase or neutral protease degrading the unwound collagen strands. The tight packing of collagen monomers in collagen fibrils or fibers enables collagenase and neutral protease to cut many native collagen molecules. As degraded collagen polypeptide chains are peeled off the collagen fibril or fiber, new binding sites for collagenase become available and the process repeated. Concomitantly, collagen degradation relaxes the extracellular matrix, exposing sites on other proteins sensitive to digestion by proteases. If these proteins directly or indirectly hold cells to the matrix, their degradation results in release of individual cells from tissue.^17^

The 3 functional forms of collagenase differ in their ability to degrade native collagen. Intact C1 (C1_116 kDa_) with 2 collagen binding domains had about a 7–10 fold higher specific collagen degrading activity (CDA U/mg protein) when compared to forms having only a single collagen binding domain (C2_114 kDa_ and C1_100 kDa_).^18^ The greater efficiency of intact C1 to degrade native collagen was indirectly shown by comparing the effectiveness of different purified enzyme mixtures manufactured by VitaCyte (VC) or Serva/Nordmark (S/N) to recover human islets. VC's mixtures containing intact C1, C2 and thermolysin gave significantly higher pre and post purification islet yields and shorter digestion times than S/N's mixtures that contained primarily truncated C1, C2 and *C. histolyticum* neutral protease (CHNP).^19^ This difference did not appear to be due to differences in the neutral protease since subsequent results showed CHNP was more effective than thermolysin in recovering islets from human pancreata.^20^

We sought to test the efficiency of intact C1 versus truncated C1 to recover islets from adult porcine pancreata when these enzymes were used with C2 and neutral protease. Recombinant intact C1 (rC1_116kDa_), intact C2 (rC2_114kDa_) or truncated C1 collagenase (rC1_100kDa_) clones were expressed in a *E. coli* strain (BL21 (DE3) pLysS) and purified from the cell lysate as described earlier.^15^ BP Protease was purified from *Paenibacillus polymyxa* culture supernatants. Pancreata were procured from retired purebred Landrace or Yorkshire X Landrace breeders (18-36 months of age) at a local abattoir. Warm ischemia times were always less than 10 minutes. The main pancreatic duct was catheterized and the organ was distended with 100 mL of ice-cold modified University of Wisconsin solution (UW) prior to transport in cold HBSS to the isolation facility. After trimming free of external fat, lymph nodes and non-pancreatic tissue, the organ was divided into the splenic and duodenal/connecting lobes such that direct comparisons between intact and truncated C1 collagenase could be made. Equivalent enzymatic target activities for all isolations were maintained at 25,000 CDA U/g trimmed tissue, 7.5 Wunsch U/g, and 12,000 FITC-BSA U/g for rC1_116kDa_/rC1_100kDa_, rC2_114kDa_ and BP Protease, respectively. Isolations with rC1_116kDa_ (n = 8) and rC1_100kDa_ (n = 8) were equally stratified across splenic and duodenal/connecting lobes to avoid confounding factors. The organ was distended with an enzyme volume equal to 1.7 X organ weight over a period of approximately 10 minutes. The pancreas was divided into 8–10 equivalent sized pieces and digestions were performed at 33°C in a Ricordi chamber. The digestion was stopped after visual analysis of samples from the digestion circuit indicated maximal islet release. The digested tissue in the circuit was diluted with room temperature Hanks Balanced Salt Solution (HBSS) and collected into ice-cold HBSS containing 20% heat-inactivated pig serum (HIPS). After three washes in HBSS-10% HIPS, digested tissue was incubated for 1 hour in modified University of Wisconsin solution. Islets were purified *via* a continuous Iodixanol (Optiprep) gradient (heavy 1.108 g/cm^3^, light 1.077 g/cm^3^) using a COBE 2991 cell processor. Islets were “bottom-loaded” by gravity and gradients were capped with 50 ml of HBSS-10% HIPS. After gradients were loaded, COBE was spun for 3 minutes at 1500 rpm. Twelve 25-ml fractions were collected and washed in cold HBSS-10% HIPS before counting.

Figure 1 shows the percentage undigested tissue, packed tissue volume per g pancreas and post-purification islet yield expressed as islet equivalents per g tissue were similar between isolations performed with either intact or truncated C1 (Figures 1a, b, c). Switch times were also similar between the isolations performed with the intact and truncated C1 enzymes, 10.3 ± 0.32 and 10.3 ± 0.76 minutes, respectively. The striking observation was the total mass of intact C1 protein required to maintain a 25,000 CDA U/g trimmed tissue target averaged 37.6 ± 2.7 mg when compared to 504.6 ± 52.5 mg for truncated C1 (Figure 1d). This ≈ 13 fold difference in mass required to achieve this targeted CDA U/g tissue reflects differences in CDA specific activity: 5,050 for truncated C1 as compared to 87,500 CDA U/mg for intact C1.

**Figure 1.**
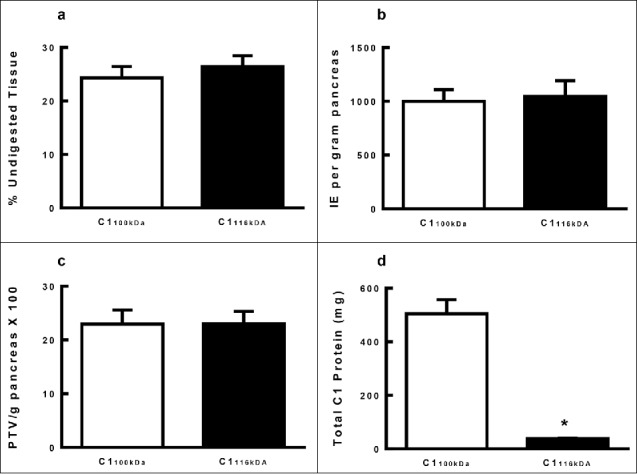
Impact of Molecular Form of Recombinant Class I Collagenase [i.e. intact (rC1_116kDa_) vs truncated protein (rC1_100 kDa_)]on Digestion of the Porcine Pancreas. Split pancreas experiment, n = 6. CDA activity was targeted at 25,000 CDA U/gram in a standardized Ricordi procedure for both molecular forms of recombinant C1. Wunsch activity was provided by recombinant C2 and maintained at 7.5 Units/gram. a, b, c, respectively, % undigested tissue, packed tissue volume (PTV) and average islet yield (IE/g) were equivalent between the two molecular forms of C1. d To match CDA activity of the rC1, approximately 13-fold higher protein concentrations were required for the C1_100 kDa_, * P < .001

These results emphasize the importance of specific CDA as a key determinant for characterizing enzyme mixtures used for cell isolation. The 25,000 CDA U/g pancreas dose was initially used at the University of Minnesota to recover pig islets from retired breeders and was adopted for isolating adult porcine islets at VitaCyte. VitaCyte's Collagenase MA product used for these isolations contained a 60% C1 (mixture of intact and truncated C1) and 40% C2 collagenase. The above experiments were performed to determine if recombinant C1 and C2 collagenase could replace the natural purified mixture of truncated and intact C1 collagenase. The unanswered question was what effect does the molecular form of rC1 on the recovery of islets from porcine pancreas?

Earlier results indicated the superiority of using intact C1 in a collagenase-protease enzyme mixtures when compared to those that contained primarily truncated C1.^19^ A subsequent report summarized results from 90 human islet isolations using 8 different enzyme mixtures. A “new enzyme mixture” composed of intact C1 and C2 mixed with S/N *C. histolyticum* neutral protease (n = 12) gave significantly shorter digestion times and higher post purification islet yields results when compared to those isolations using S/N GMP grade enzymes (n = 20) or S/N Premium Grade enzymes (n = 5).^20^ An earlier report from Brandhorst et al. also noted the decrease in human islet yield when the S/N enzymes were used in place of Liberase HI^21^, a product shown to contain both intact and truncated C1.^19^

A recent report by Brandhorst, et al., showed differences in the efficiency of intact and truncated C1 for recovery of islets from human tissue.^22^ Intact C1 was more effective in islet recovery than truncated C1 when enzyme mixtures contained C2 but no supplemental protease. In contrast, there was no difference in islet recoveries when the enzyme mixtures above contained supplemental neutral protease activity. The authors suggested that differences in the efficiencies of C1 molecular forms to recover human islets can be overcome if supplemental protease is used in the enzyme mixture. Their conclusion is not supported by earlier studies which showed a difference in the performance of enzyme mixtures that contained primarily intact or truncated C1 with C2 and an optimal dose of protease.^19,20^ Also, these authors did not cite earlier literature that showed endogenous pancreatic protease activity is sufficient to replace exogeneous neutral protease for islet isolation.^23^ Endogenous protease activity increases with prolonged cold ischemia time.^17,23,24^ There is also potential to increase endogenous protease activity by having contaminating clostripain present in purified collagenase convert pancreatic proenzymes into active serine proteases.^17^

Discrepancies regarding the importance of intact C1 in isolating islets remains puzzling, but points to the complexity of human pancreas digestion and strongly suggest a number of factors, including enzymes used to isolate islets, may influence the outcome. The conclusion from the present report may provide additional guidance when selecting collagenase products to isolate mammalian islets. The most efficient collagenase preparations to release cells from tissue will contain primarily intact C1, C2, and neutral protease. This translates into lower amounts of collagenase used for islet isolation when compared to enzyme mixtures that contain primarily truncated C1. This will be relevant for development of robust islet isolation processes to generate porcine or human therapeutic islet products. Validation of enzymes used in the isolation procedure is a critical process parameter where acceptable working ranges for enzyme activities should be set to control the manufacturing procedure. Assessment of a functional collagenase activity (i.e., CDA) using the most efficient forms of collagenase will be the expected norm to ensure cost effective manufacture of a functional therapeutic product.
